# Sry delivery to the adrenal medulla increases blood pressure and adrenal medullary tyrosine hydroxylase of normotensive WKY rats

**DOI:** 10.1186/1471-2261-7-6

**Published:** 2007-02-26

**Authors:** Daniel Ely, Amy Milsted, Jason Bertram, Mat Ciotti, Gail Dunphy, Monte E Turner

**Affiliations:** 1 Department of Biology, University of Akron, Akron, OH 44325 USA

## Abstract

**Background:**

Our laboratory has shown that a locus on the SHR Y chromosome increases blood pressure (BP) in the SHR rat and in WKY rats that had the SHR Y chromosome locus crossed into their genome (SHR/y rat). A potential candidate for this Y chromosome hypertension locus is Sry, a gene that encodes a transcription factor that is responsible for testes development and the Sry protein may affect other target genes.

**Methods:**

The following study examined if exogenous Sry would elevate adrenal Th, adrenal catecholamines, plasma catecholamines and blood pressure. We delivered 10 μg of either the expression construct, Sry1/pcDNA 3.1, or control vector into the adrenal medulla of WKY rats by electroporation. Blood pressure was measured by the tail cuff technique and Th and catecholamines by HPLC with electrochemical detection.

**Results:**

In the animals receiving Sry there were significant increases after 3 weeks in resting plasma NE (57%) and adrenal Th content (49%) compared to vector controls. BP was 30 mmHg higher in Sry injected animals (160 mmHg, p < .05) compared to vector controls (130 mmHg) after 2–3 weeks. Histological analysis showed that the electroporation procedure did not produce morphological damage.

**Conclusion:**

These results provide continued support that Sry is a candidate gene for hypertension. Also, these results are consistent with a role for Sry in increasing BP by directly or indirectly activating sympathetic nervous system activity.

## Background

We have shown previously that there is a locus on the SHR Y chromosome that increases blood pressure (BP) about 20–25 mmHg [1,2]. Backcrosses of the SHR Y chromosome into WKY rats show a significant Y chromosome BP increase of 20 mmHg [3]. Further studies showed that the SHR Y chromosome increased several indices of SNS activity [4]. We demonstrated that renal norepinephrine (NE) turnover rate is higher by 100% [5] and renal NE content is 44% higher in males with the Y chromosome from an SHR [5]. Our recent studies indicate that a candidate gene for this SNS and BP effect is Sry, a transcription factor on the Y chromosome that is the testis determining factor [6]. Sry expression has been reported in testis, the brain and in additional tissues that have BP relevance in adult humans and rodents [7-9]. Recently, we demonstrated that Sry increased tyrosine hydroxylase (Th) promoter activity in transfected PC12 cells [6] although much of the effect was indirect. Th catalyzes the conversion of L-tyrosine to L-dihydroxyphenylalanine (L-DOPA) which ultimately leads to the formation of dopamine and NE. Th is the rate limiting enzyme in the catecholamine biosynthesis pathway; thus regulation of Th by Sry is a potential pathway for Sry to affect BP. Since the effect of Sry in adult males is not understood there may be a variety of mechanisms raising BP by Sry.

Based on these findings we developed the following hypothesis: delivery of exogenous Sry to the adrenal medulla of a WKY rat increases Th activity in the adrenal medulla, thereby leading to elevated plasma NE and increased BP. To address this hypothesis, we added exogenous SHR Sry gene sequences to the adrenal medulla of a normotensive WKY rat using gene delivery by electroporation to see if there was an increase in Th, NE and BP. While the electroporation technique of gene delivery was developed for use primarily in cell culture, it has also been successfully used in various tissues in the whole animal. Procedures using DNA injection coupled with electrostimulation increased efficiency over both direct injection into skeletal muscle (by 100×–1000×) [10] and in the use of adenoviral techniques for gene delivery to gliomas (100× increase) [11]. In other studies, delivery of exogenous genes produced physiological responses in nephrectomized rats, where rat erythropoietin injection corrected anemia [12], and in another study raised the hematocrit from 47% to 80% [13]. Tsuji et al examined the efficiency of intrarenal injection of DNA followed by in vivo electroporation and found that mesangial cells were transfected and no histological damage was observed [14]. So the literature supports the notion that delivery of exogenous DNA can cause physiological changes. Following electroporation of Sry into the adrenal medulla of WKY rats, we show that blood pressure and sympathetic nervous system indices increase.

## Methods

Adult WKY males were used for all studies (n = 24). All animal protocols were approved by the Institutional Animal Care and Use Committee of the University of Akron (IACUC proposal # 03–03A). The experimental design consisted of two studies. The first study involved the addition of exogenous Sry or control vector to the adrenal medulla of normotensive WKY rats. An Sry1 expression construct, Sry1/pcDNA3.1(-), was prepared by cloning bp #1–1048 of SHR Sry1 (GenBank accession number AF274872) of pcDNA3.1(-) (Invitrogen) as described previously [6]. This includes the complete SHR Sry1 coding sequence (bp#11–520 of GenBank accession number, AF274872). Either 10 μg of Sry1/pcDNA3.1(-) (n = 6) or 10 μg of control pcDNA3.1(-) vector without Sry1 sequences (n = 6) was injected into the left adrenal medulla of 6 WKY adult male rats using pentothal as an anesthetic (50 mg/kg,ip). Sry or empty vector control was transfected by injection (Hamilton 10 μL syringe with 30 g needle) followed by electroporation into the adrenal medulla. A mark was placed on the needle to insure proper depth to the medulla. A drop of glycerol was placed on the injection site before injection to prevent backflow.

In a second study, in order to verify that the electroporation procedure did not cause adrenal damage, the histology of the adrenal gland was examined after electroporation. After stimulation the incision was closed and the animal allowed to recover. The electroporation protocol was followed and adult male WKY were injected with Sry1/pcDNA3.1(-) (n = 6) or empty vector (n = 6) and electroporated in order to examine the tissue for damage. A control group of WKY adrenal glands (n = 6) that was not injected or electroporated was also examined.

### Electroporation

Each tissue has different optimal electroporation parameters that have been described for gene delivery [11]. Based on previous research and our own pilot tests we performed electroporation in a pulsatile fashion immediately after injection. Tweezer electrodes (BTX Tweezertrodes Model #522) connected to an electrostimulator (ElectroCellManipulator™, ECM^® ^830 ELECTROPORATION PROTOCOL, BTX, a division of Genentronics), were placed on opposite sides of the adrenal gland and 20 bipolar electrical pulses administered, 200 volts, each lasting 20 msec, at 1000 Hz. After stimulation the incision was closed and the animal allowed to recover.

### Blood Pressure

Systolic blood pressure was measured in conscious animals by the tail cuff technique weekly for 3 weeks [1,3,15]. Briefly, the animals are warmed in a heating chamber (37°C) for 20–30 minutes to vasodilate and 5 consecutive pressures are recorded which takes less than 3 minutes.

### Catecholamine Analysis

Levels of adrenal Th and catecholamines, as well as plasma catecholamines, were measured 3 weeks after electroporation. Th and catecholamines were measured by HPLC with electrochemical detection (Waters 2465, Milford, MA). For catecholamines the mobile phase consisted of citric acid (35 mM), sodium acetate (90 mM), octyl sodium sulfate (690 μM), EDTA (130 μM) and 10% methanol, pH adjusted to 4.7. For L-Dopa analysis the same amount as above were used for the mobile phase except octyl sodium sulfate was increased to 920 μM and no methanol was added. This was due to the rapid release of L-Dopa from the column. The pump (Waters 1515) ran at 1.4 ml/min, 50 ul was injected onto the column (Waters column, 4.6 × 150 mm, C18, 5 μ, preceded by a guard column (LC-18, Supelco, Bellfonte, PA). The samples were maintained at 10°C using the 717 plus autosampler (Waters, Milford, MA). Catecholamines were extracted from plasma by placing 300 μl of plasma into 1 ml of 1 M Tris buffer, pH 8.7 (consisting of 1 M Tris,10 mM sodium metabisulfite, 20 mM EDTA (Sigma) with 25 mg of LC-alumina A (Supelco, Bellfonte, PA) and vigorously vortexing for 10 minutes. The samples were then washed 3× with 1 ml of wash solution (0.2% TRIS-EDTA, Sigma) and mixed 3 minutes followed by aspiration of the supernatant after each wash. The last step was the addition of 400 μl of 100 mM perchloric acid and vortexing for 5 minutes and removal of the supernatant for injection into the HPLC. The Th activity was calculated by measuring L-DOPA formed per milligram tissue per minute. Adrenal glands were homogenized in 500 μl of 0.25 M sucrose. The homogenate (100 μl) was added to both a blank tube and a reaction tube. The chemical reaction was based on procedures developed by Nagatsu [16] and modified by Hooper et al [17] and Kumai et al. [18] followed by extraction using the same method as for catecholamines.

### Adrenal Gland Morphology

In the second study the adrenal glands were removed, stored in 10% buffered formalin, dehydrated in a tissue processor (Tissue-Tek, Miles Inc., Elkhart, IN) and paraffin embedded (Paraplast Plus, Fisher Sci). Sections were cut at 6 μ and stained with hemotoxylin and eosin (Richard Allan scientific) to assess the tissue for potential gene delivery-electroporation injury.

BP and catecholamine levels were analyzed using Student's t-tests; means and standard error of the means are reported; significance was assumed if p < .05.

## Results

**Figure 1:**
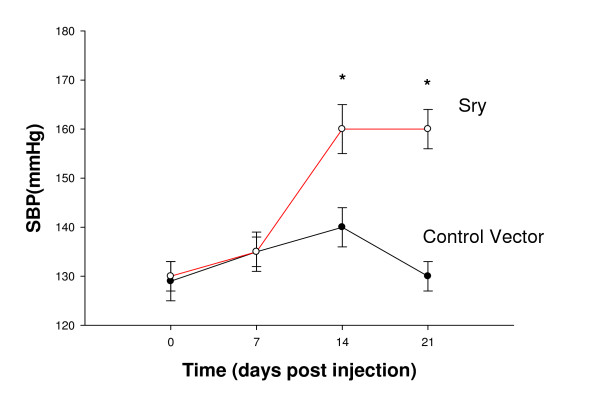
Systolic blood pressure (SBP) in the vector controls vs Sry injected at baseline weekly for 3 weeks (means, =/-s.e.m., * = p < .05).

**Figure 2:**
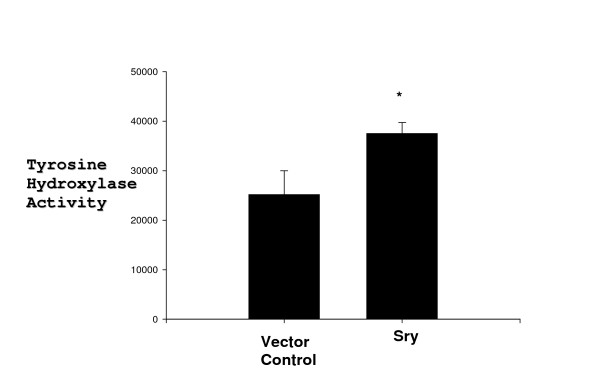
Tyrosine hydroxylase activity (measured as L-DOPA formed per minute per mg tissue, fm/min/mg) in adrenal gland 3 weeks after plasmid delivery (means, +/-s.e.m., * p <.05).

**Figure 3:**
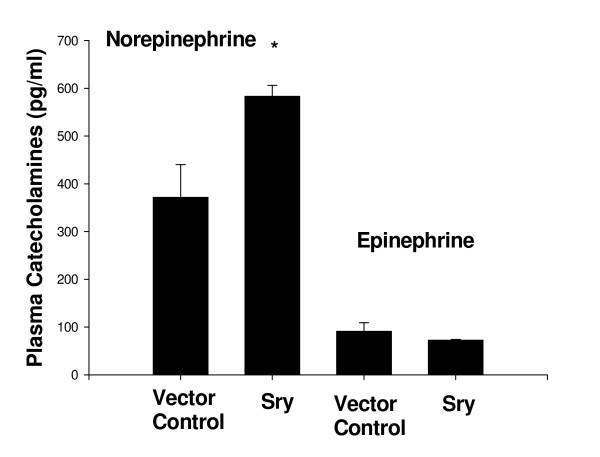
Plasma catecholamines 3 weeks after plasmid delivery (means +/-s.e.m., * = p < .05).

Figure 1 shows the weekly systolic blood pressure (SBP) before and during the 3 weeks after delivery of either Sry or the empty control vector to the adrenal medulla. There was a significant increase in BP 2–3 weeks after Sry delivery as compared to control vector, 160 mmHg and 160 mmHg vs. 140 mmHg and 130 mmHg, respectively (p < .05). Figure 2 shows that 3 weeks after Sry delivery to the adrenal medulla, Th was significantly increased compared to the vector control (37,494 vs. 25,181 fmol/min/mg, p = .017). these values were comparable to Kumai's values in male WKY (16,670 fmol/mg/min converted to the units we used) [19]. Figure 3 shows that 3 weeks after Sry delivery, plasma NE was significantly increased compared to the vector control group (584 vs. 372 pg/ml, p =.026); however, there was no change in plasma epinephrine level between the two groups. With regard to adrenal catecholamine content there were no significant differences between vector control or Sry groups after 3 weeks (NE:241+/-102 vs. 247+/-33 ng/mg, E: 1090+/-404 vs. 1220+/-128 ng/mg, respectively). Also the plasma NE values of the two electroporated groups was similar to previous values in WKY reported from our lab suggesting that the electroporation procedure did not damage the adrenal medulla[20].

**Figure 4:**
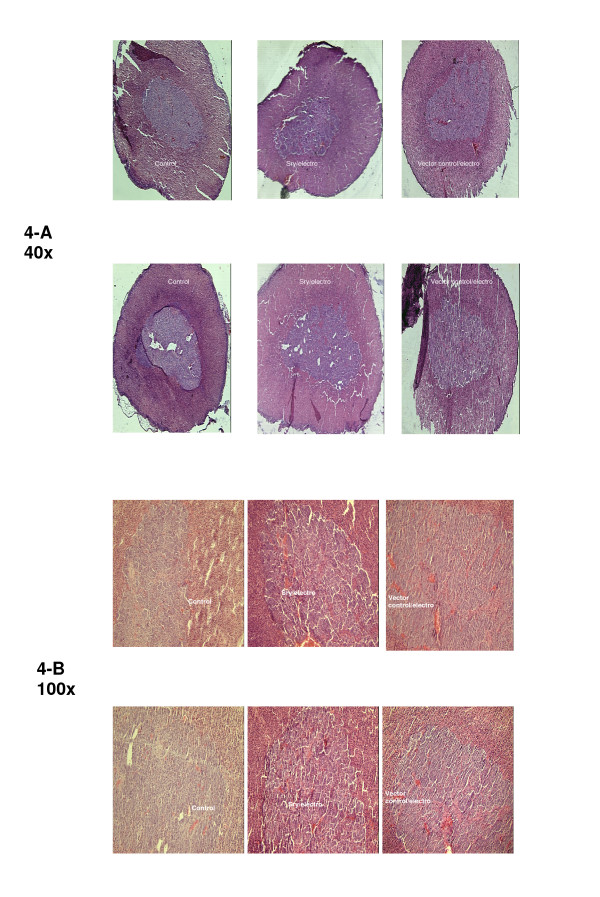
Adrenal gland morphology is unchanged by the electroporation protocol. A panel-40×, B panel-100×, of representative animals that had nothing done-(left panels, control), had Sry delivered by electroporation (middle panels, Sry/electro) or empty vector delivered (right panels). The lighter color purple in the middle of the tissue is the medulla. The holes or cracks in the medulla are not defects but tears in the tissue from processing. Gene or empty vector delivery did not disrupt tissue morphology.

Histological examination of the adrenal medulla did not show any morphological differences between vector control, Sry delivered or controls without any procedure (Figure 4). There was concern that electroporation may damage medullary cells but the morphology of the two electroporated groups were similar to the non-elctroporated controls.

## Discussion

Our main finding is the potential role for Sry in elevating BP. The time scale of the BP response is consistent with the responses of other genes delivered by electroporation which is about 3 weeks for peak effect [21]. The lack of BP increase with plasmid control vector demonstrates that the BP increase was due specifically to the addition of Sry sequences, not to electroporation itself or an effect of the plasmid sequences.

Adding exogenous Sry DNA from SHR to the adrenal gland of WKY rats increased BP by 30 mmHg, comparable to the 20 mmHg effect we see when the SHR Y chromosome is added by genetic crosses in our Y chromosome consomic strains. Typical effects on BP attributed to other candidate genes or loci are small or equal to the 30 mmHg effect we find after SHR Sry delivery to WKY adrenal gland. For instance, in an review of genetic hypertension in rats, Rapp shows with linkage analysis that in the Dahl salt sensitive rat there are QTLs on chromosome 9 that are associated with blood pressure increases of 5, 8, 12, 13, 18, 21 and 26 mmHg [22]. However, these are large regions with many genes that may or may not have physiological functions that influence blood pressure.

The identification of a Y chromosome effect on blood pressure from a cross between SHR and WKY was the equivalent to finding a QTL on an autosomal chromosome. In this case though, the QTL included all of the genes on the Y chromosome except those in the pseudoautosomal region. A major difference from an autosomal QTL is the inability to decrease the size of the Y chromosome region using crossing over. Because the mammalian Y chromosome contains very few genes considering its size, we were able to eliminate most of the Y chromosome genes from consideration. The Sry locus was first identified as a logical candidate because of its potential to modulate the sympathetic nervous system (SNS) since the related Sox loci are responsible for nervous system development. Following this line of reasoning we have shown that normotensive WKY animals that have a Y chromosome from a SHR father had elevated SNS indices [4,5]. Also we have sympathectomized neonatal males with the SHR Y chromosme loci and eliminated the rise in BP [23]. We evaluated potential effects of Sry on the tyrosine hydroxylase promoter. *In vitro *studies co-transfecting an Sry expression vector and a tyrosine hydroxylase promoter controlled reporter gene, confirmed that Sry directly or indirectly increased tyrosine hydroxylase promoter activity [6]. Although this demonstrated that Sry has an effect on the tyrosine hydroxylase promoter in cultured cells, that doesn't mean that in animals it would necessarily affect blood pressure. In the current experiments, we have continued with Sry as a candidate gene but have moved from cell culture to animal experiments.

We provide evidence here that part of the BP rise may be due to Th in the adrenal medulla. This does not exclude other potential targets of Sry, such as, the renin-angiotensin system (RAS) and testosterone. We cannot eliminate the RAS as a player in the Sry story so further investigation is needed. However, we did examine plasma renin levels after Sry delivery to the kidney in WKY males and there was no plasma rennin activity difference from vector controls (unpublished data). With regards to the possibility that the adrenal cortex may have been involved due to the medullary injections we have not directly studied potential steroid targets of Sry, such as, adrenal aldosterone, glucocorticoids and testosterone. However, we have examined renal function and found normal sodium excretion and creatinine and albumin clearance (unpublished data). One would expect to see altered sodium excretion if Sry modulated plasma aldosterone levels. Although an indirect measure of a glucocorticoid effect, we have measured plasma and urinary glucose after Sry delivery to the kidney and found no effect compared to controls. We have evidence that plasma corticosterone in WKY controls was not different than that in WKY males with the SHR Y chromosome [24]. This suggests that the SHR Y locus does not endogenously alter corticosterone, however, it is possible that Sry delivered to the adrenal medulla could have a corticosterone effect. Testosterone most likely is an important factor promoting the BP rise since our previous studies have shown that the SHR Y chromosome produces an earlier rise in plasma testosterone compared to WKY males or SHR males with the WKY Y chromosome [25]. However, we have not yet made the direct connection between Sry and a rise in plasma testosterone. Sry could have an influence in hypothalamic and pituitary release of gonadotropins. This is an area for future study. A piece of evidence that would suggest that adrenal testosterone was not effected by adrenal medullary injection of Sry is that in castrated WKY males we do not detect any plasma testosterone even up to 10 weeks after castration which is enough time for adrenal compensation to take place [26]. Still it is possible that stringent stimulation of the medulla could release cortical testosterone. Indeed, a provocative area of research is the crosstalk between the adrenal cortex and medulla. A decrease in Th in chromaffin cells can decrease adrenal cortical function and conversely, an elevation in plasma catecholamines can elevate plasma aldosterone and glucocorticoids [27].

Elevated Th and/or catecholamine levels are established physiological characteristics of many hypertensive animal models and human studies [28-31]. We have shown that the Y chromosome from a SHR male when backcrossed to a normotensive WKY female increased SNS indices [4] and maintained an increase in BP of about 20 mmHg even after 11 generations of backcrossing sons to WKY females (SHR/y) [3]. We also showed that in SHR/y compared to WKY, renal and heart NE turnover is higher, although the NE organ content did not change [5]. In fact, the kidney norepinephrine turnover rate in the SHR/y males was the same as in SHR males suggesting that the catecholamine pathway, at least in the kidney, is significantly influenced by the Y chromosome [5].

Our results showed that although Th and plasma NE were elevated after Sry delivery, total adrenal NE content was not elevated. One explanation for this could be that the elevated NE release into the plasma may have depleted the NE vesicle storage content. Multiple and complex mechanisms contribute to Th activity and NE synthesis during SNS activation. Short term mechanisms include feedback inhibition and enzyme phosphorylation. Long term mechanisms include changes in Th synthesis [32]. There appears to be an uncoupling of the tissue NE content, the activity of Th and the release of NE. Two storage pools of NE exist in both the SNS nerve terminals and in the adrenal medulla: a small readily releasable pool of newly synthesized NE and a large reserve pool in long term storage [33]. For example, studies in rats show a 35% reduction in Th activity at all ages studied (5, 12, 22 weeks) while plasma NE was 3–4× higher in SHR compared to WKY rats [34]. Therefore, NE storage, cytoplasmic pools and plasma levels need not be in a linear relationship. Also, plasma E did not increase like NE did after Sry administration. An explanation for this may be that the Sry transcript is working on the enzyme, Th, and not on the enzyme that produces E, PNMT. Since both end products exist in the adrenal medulla there is not necessarily a 1/1 relationship with NE/E.

Our results support the notion that SHR Sry increases adrenal Th, leading to increased plasma NE and increased peripheral vasoconstriction resulting in increased BP in a WKY normotensive animal. Indeed, BP increased 30 mmHg after 3 weeks and adrenal Th activity increased 49% and plasma NE increased 57%. Further support for the Th-BP connection is provided by Kumai's study showing that antisense Th decreased both Th and BP in SHR [31]. Also Kumai et al. suggest that androgens contribute to the development and maintenance of hypertension in SHR via sustained enhancement of Th synthesis in the adrenal medulla, leading to increased epinephrine and norepinephrine levels [19]. Further mechanistic studies are needed to determine if Sry induced increase in Th is associated with enhanced adrenal release of NE, altered reuptake and other target organs, such as the adrenal cortex.

## Conclusion

In conclusion, we were the first group to show a Y chromosome BP effect and our current findings are the first to support SHR Sry as a candidate gene on the Y chromosome. Typical effects on BP attributed to other candidate genes are small compared to the 30 mmHg effect we find after SHR Sry delivery to WKY adrenal gland. The electroporation results demonstrate the potential for Sry to have a hypertensive effect. We have suggested a mechanism operating through the catecholamine pathway and increased sympathetic nervous system activity which is supported by our previous research. However, since Sry is expressed in several different tissues that could influence blood pressure, such as, the brain, heart and kidney, there may be more than one mechanism and even secondary effects of Sry on blood pressure especially through neuroendocrine pathways. Although this is the first Y chromosome locus to have hypertensive potential demonstrated, this result is not sufficient to conclude that Sry is either solely or directly responsible for the hypertensive effect of the SHR Y chromosome. The key to the hypertensive effect of the SHR Y chromosome is that it was discovered in a comparison to the normotensive WKY Y chromosome. Further studies are needed to examine expression and sequence differences between the SHR and WKY Sry loci to conclude that Sry is the hypertensive locus on the SHR Y chromosome.

## Competing interests

The author(s) declare that they have no competing interests.

## Authors' contributions

DE, AM, and MT designed and directed studies, were involved in the analysis of the data, and writing of the manuscript. JB, MC and GD performed the experiments and GD ran the assays. All authors have read and approved manuscript.

## Pre-publication history

The pre-publication history for this paper can be accessed here:
